# Supplementary material for: Meta-Analysis and Gene Set Enrichment Relative to ER Status Reveal Elevated Activity of MYC and E2F in the “Basal” Breast Cancer Subgroup
Source: PLoS One. 2009 Mar 9;4(3):e4710. doi: 10.1371/journal.pone.0004710 (PMC2650420; doi:10.1371/journal.pone.0004710)
Supplement: Dataset S1 — GSEA results from first screen. The file contains a zipped archive of the HTML results of the second GSEA screen. Reports for enriched or depleted datasets may be accessed from the “index.html” link within the my_analysis.GseaPreranked.1219975950625.rpt.zip ZIP archive. Enrichment plots are available for each of the 50 most enriched and depleted gene sets. (13.32 MB ZIP) [file pone.0004710.s001.zip › my_analysis.GseaPreranked.1219975950625.rpt.zip!//GAY_YY1_DN.html]

Details for gene set GAY\_YY1\_DN[GSEA]

|  || Dataset | zSco.ERneg.to.ERpos\_collapsed |
| Phenotype | NoPhenotypeAvailable |
| Upregulated in class | na\_pos |
| GeneSet | GAY\_YY1\_DN |
| Enrichment Score (ES) | 0.48451197 |
| Normalized Enrichment Score (NES) | 2.2100868 |
| Nominal p-value | 0.0 |
| FDR q-value | 0.0 |
| FWER p-Value | 0.0 |
Table: GSEA Results Summary

  

Fig 1: Enrichment plot: GAY\_YY1\_DN      
 Profile of the Running ES Score & Positions of GeneSet Members on the Rank Ordered List

  

| PROBE | GENE SYMBOL | GENE\_TITLE | RANK IN GENE LIST | RANK METRIC SCORE | RUNNING ES | CORE ENRICHMENT || 1 | TTK |  |  | 11 | 7.059 | 0.0128 | Yes |
| 2 | FOXM1 |  |  | 21 | 6.760 | 0.0251 | Yes |
| 3 | MCM5 |  |  | 31 | 6.533 | 0.0370 | Yes |
| 4 | BUB1 |  |  | 41 | 6.445 | 0.0488 | Yes |
| 5 | CENPA |  |  | 51 | 6.315 | 0.0603 | Yes |
| 6 | CCNA2 |  |  | 52 | 6.297 | 0.0724 | Yes |
| 7 | NASP |  |  | 59 | 6.201 | 0.0839 | Yes |
| 8 | AURKB |  |  | 76 | 6.038 | 0.0944 | Yes |
| 9 | ANP32E |  |  | 84 | 5.988 | 0.1054 | Yes |
| 10 | TRIP13 |  |  | 96 | 5.890 | 0.1159 | Yes |
| 11 | MKI67 |  |  | 98 | 5.871 | 0.1272 | Yes |
| 12 | KIF2C |  |  | 124 | 5.703 | 0.1363 | Yes |
| 13 | CYP7B1 |  |  | 131 | 5.690 | 0.1468 | Yes |
| 14 | RIPK4 |  |  | 150 | 5.576 | 0.1562 | Yes |
| 15 | MCM4 |  |  | 180 | 5.436 | 0.1644 | Yes |
| 16 | MCM6 |  |  | 198 | 5.317 | 0.1734 | Yes |
| 17 | MTHFD2 |  |  | 205 | 5.307 | 0.1832 | Yes |
| 18 | EXO1 |  |  | 218 | 5.239 | 0.1924 | Yes |
| 19 | BIRC5 |  |  | 229 | 5.191 | 0.2016 | Yes |
| 20 | RAD51AP1 |  |  | 267 | 5.028 | 0.2085 | Yes |
| 21 | CENPF |  |  | 268 | 5.028 | 0.2182 | Yes |
| 22 | BUB1B |  |  | 271 | 5.016 | 0.2277 | Yes |
| 23 | MCM7 |  |  | 289 | 4.970 | 0.2360 | Yes |
| 24 | GJB3 |  |  | 309 | 4.910 | 0.2440 | Yes |
| 25 | STMN1 |  |  | 310 | 4.907 | 0.2535 | Yes |
| 26 | KIF20A |  |  | 318 | 4.874 | 0.2624 | Yes |
| 27 | CDCA3 |  |  | 321 | 4.868 | 0.2716 | Yes |
| 28 | FOLH1 |  |  | 343 | 4.822 | 0.2793 | Yes |
| 29 | PTPN2 |  |  | 353 | 4.783 | 0.2878 | Yes |
| 30 | ASPM |  |  | 359 | 4.767 | 0.2967 | Yes |
| 31 | RFC4 |  |  | 370 | 4.741 | 0.3050 | Yes |
| 32 | CCNB2 |  |  | 431 | 4.589 | 0.3093 | Yes |
| 33 | ZRF1 |  |  | 432 | 4.585 | 0.3182 | Yes |
| 34 | AURKA |  |  | 460 | 4.483 | 0.3247 | Yes |
| 35 | MAD2L1 |  |  | 477 | 4.446 | 0.3321 | Yes |
| 36 | CXCL5 |  |  | 483 | 4.399 | 0.3402 | Yes |
| 37 | PLK4 |  |  | 524 | 4.292 | 0.3454 | Yes |
| 38 | CHST2 |  |  | 530 | 4.281 | 0.3533 | Yes |
| 39 | PRC1 |  |  | 543 | 4.253 | 0.3606 | Yes |
| 40 | PDGFRA |  |  | 556 | 4.224 | 0.3678 | Yes |
| 41 | SMC2 |  |  | 651 | 4.028 | 0.3684 | Yes |
| 42 | MCM3 |  |  | 656 | 4.019 | 0.3759 | Yes |
| 43 | PCOLCE2 |  |  | 657 | 4.019 | 0.3836 | Yes |
| 44 | TACC3 |  |  | 669 | 3.995 | 0.3905 | Yes |
| 45 | KIF11 |  |  | 713 | 3.909 | 0.3948 | Yes |
| 46 | FEN1 |  |  | 754 | 3.838 | 0.3991 | Yes |
| 47 | PRDX4 |  |  | 761 | 3.821 | 0.4060 | Yes |
| 48 | CENPE |  |  | 808 | 3.766 | 0.4098 | Yes |
| 49 | MSH2 |  |  | 819 | 3.748 | 0.4162 | Yes |
| 50 | MTCH2 |  |  | 946 | 3.560 | 0.4135 | Yes |
| 51 | FDPS |  |  | 949 | 3.555 | 0.4202 | Yes |
| 52 | SHCBP1 |  |  | 986 | 3.501 | 0.4242 | Yes |
| 53 | PLK1 |  |  | 988 | 3.499 | 0.4309 | Yes |
| 54 | PLSCR1 |  |  | 1002 | 3.481 | 0.4366 | Yes |
| 55 | KIF23 |  |  | 1030 | 3.442 | 0.4412 | Yes |
| 56 | MRPL19 |  |  | 1061 | 3.401 | 0.4455 | Yes |
| 57 | UNG |  |  | 1070 | 3.390 | 0.4514 | Yes |
| 58 | SUV39H2 |  |  | 1110 | 3.349 | 0.4549 | Yes |
| 59 | HMGB3 |  |  | 1128 | 3.330 | 0.4600 | Yes |
| 60 | HELLS |  |  | 1382 | 3.057 | 0.4466 | Yes |
| 61 | RAB27A |  |  | 1438 | 3.011 | 0.4482 | Yes |
| 62 | TNFRSF1B |  |  | 1474 | 2.970 | 0.4512 | Yes |
| 63 | PIGA |  |  | 1546 | 2.905 | 0.4514 | Yes |
| 64 | SLC25A5 |  |  | 1599 | 2.850 | 0.4530 | Yes |
| 65 | HMMR |  |  | 1608 | 2.843 | 0.4578 | Yes |
| 66 | CCNB1 |  |  | 1626 | 2.823 | 0.4620 | Yes |
| 67 | CCL2 |  |  | 1664 | 2.791 | 0.4645 | Yes |
| 68 | TGFBI |  |  | 1668 | 2.786 | 0.4697 | Yes |
| 69 | CDC25C |  |  | 1794 | 2.700 | 0.4654 | Yes |
| 70 | SQLE |  |  | 1821 | 2.677 | 0.4685 | Yes |
| 71 | SCARB1 |  |  | 1828 | 2.674 | 0.4732 | Yes |
| 72 | D4S234E |  |  | 1832 | 2.669 | 0.4782 | Yes |
| 73 | ADCY7 |  |  | 1904 | 2.604 | 0.4778 | Yes |
| 74 | CRABP1 |  |  | 1975 | 2.553 | 0.4773 | Yes |
| 75 | LGR5 |  |  | 2001 | 2.529 | 0.4803 | Yes |
| 76 | PRELP |  |  | 2052 | 2.482 | 0.4813 | Yes |
| 77 | H2AFZ |  |  | 2105 | 2.447 | 0.4820 | Yes |
| 78 | SOD3 |  |  | 2135 | 2.429 | 0.4845 | Yes |
| 79 | DUSP9 |  |  | 2335 | 2.300 | 0.4738 | No |
| 80 | BCAT1 |  |  | 2344 | 2.290 | 0.4776 | No |
| 81 | ACAT2 |  |  | 2472 | 2.215 | 0.4721 | No |
| 82 | PTDSR |  |  | 2493 | 2.202 | 0.4749 | No |
| 83 | HMGB1 |  |  | 2520 | 2.191 | 0.4771 | No |
| 84 | CDT1 |  |  | 2522 | 2.190 | 0.4813 | No |
| 85 | HMGCS1 |  |  | 2577 | 2.158 | 0.4813 | No |
| 86 | GZMA |  |  | 2627 | 2.130 | 0.4817 | No |
| 87 | TK1 |  |  | 2730 | 2.076 | 0.4779 | No |
| 88 | NSDHL |  |  | 2748 | 2.067 | 0.4806 | No |
| 89 | LMNB1 |  |  | 2764 | 2.060 | 0.4834 | No |
| 90 | XRCC4 |  |  | 2918 | 1.977 | 0.4755 | No |
| 91 | CDC6 |  |  | 3185 | 1.847 | 0.4588 | No |
| 92 | TMEM45A |  |  | 3492 | 1.713 | 0.4388 | No |
| 93 | ELOVL6 |  |  | 3573 | 1.675 | 0.4359 | No |
| 94 | SFTPC |  |  | 3603 | 1.662 | 0.4369 | No |
| 95 | PLCB1 |  |  | 3608 | 1.660 | 0.4398 | No |
| 96 | NR1H4 |  |  | 3695 | 1.621 | 0.4363 | No |
| 97 | FZD3 |  |  | 3728 | 1.612 | 0.4370 | No |
| 98 | TOP2A |  |  | 3735 | 1.610 | 0.4396 | No |
| 99 | TDRD1 |  |  | 3853 | 1.570 | 0.4337 | No |
| 100 | HMGB2 |  |  | 3883 | 1.558 | 0.4345 | No |
| 101 | PRPS1 |  |  | 3889 | 1.556 | 0.4372 | No |
| 102 | POLA1 |  |  | 3967 | 1.525 | 0.4342 | No |
| 103 | FGF7 |  |  | 4038 | 1.499 | 0.4318 | No |
| 104 | RACGAP1 |  |  | 4097 | 1.475 | 0.4302 | No |
| 105 | MYO1B |  |  | 4100 | 1.473 | 0.4329 | No |
| 106 | ALDH18A1 |  |  | 4138 | 1.457 | 0.4329 | No |
| 107 | IL1RL1 |  |  | 4197 | 1.433 | 0.4312 | No |
| 108 | RPS6KA4 |  |  | 4307 | 1.389 | 0.4256 | No |
| 109 | FARS2 |  |  | 4350 | 1.372 | 0.4250 | No |
| 110 | SPP1 |  |  | 4426 | 1.346 | 0.4219 | No |
| 111 | HSPB7 |  |  | 4492 | 1.321 | 0.4195 | No |
| 112 | SLC4A10 |  |  | 4600 | 1.286 | 0.4138 | No |
| 113 | FCGR2B |  |  | 4628 | 1.278 | 0.4142 | No |
| 114 | LIG1 |  |  | 4659 | 1.266 | 0.4143 | No |
| 115 | PTGS1 |  |  | 4669 | 1.264 | 0.4161 | No |
| 116 | API5 |  |  | 4676 | 1.260 | 0.4181 | No |
| 117 | PLA2G1B |  |  | 4748 | 1.238 | 0.4150 | No |
| 118 | AVPR1A |  |  | 4755 | 1.235 | 0.4169 | No |
| 119 | ANK1 |  |  | 4791 | 1.226 | 0.4166 | No |
| 120 | TNFSF11 |  |  | 4945 | 1.174 | 0.4072 | No |
| 121 | HAS2 |  |  | 5204 | 1.079 | 0.3896 | No |
| 122 | LDLR |  |  | 5398 | 1.012 | 0.3768 | No |
| 123 | PTGDS |  |  | 5528 | 0.965 | 0.3688 | No |
| 124 | MCHR1 |  |  | 5578 | 0.951 | 0.3669 | No |
| 125 | CCL7 |  |  | 5627 | 0.937 | 0.3651 | No |
| 126 | TTF1 |  |  | 5704 | 0.910 | 0.3610 | No |
| 127 | GK2 |  |  | 5735 | 0.901 | 0.3605 | No |
| 128 | KHDRBS1 |  |  | 5819 | 0.872 | 0.3558 | No |
| 129 | STEAP1 |  |  | 5866 | 0.855 | 0.3540 | No |
| 130 | IL1RAPL2 |  |  | 5875 | 0.852 | 0.3550 | No |
| 131 | INCENP |  |  | 5907 | 0.845 | 0.3543 | No |
| 132 | GALR1 |  |  | 5958 | 0.830 | 0.3521 | No |
| 133 | CSH1 |  |  | 5969 | 0.824 | 0.3529 | No |
| 134 | SPAG5 |  |  | 6030 | 0.800 | 0.3498 | No |
| 135 | ITGA4 |  |  | 6055 | 0.794 | 0.3495 | No |
| 136 | KLRA1 |  |  | 6095 | 0.783 | 0.3481 | No |
| 137 | FADS2 |  |  | 6236 | 0.735 | 0.3388 | No |
| 138 | VAV1 |  |  | 6308 | 0.714 | 0.3348 | No |
| 139 | GPR65 |  |  | 6340 | 0.705 | 0.3338 | No |
| 140 | IDI1 |  |  | 6517 | 0.645 | 0.3216 | No |
| 141 | POLH |  |  | 6522 | 0.642 | 0.3225 | No |
| 142 | TGFBR1 |  |  | 6636 | 0.611 | 0.3151 | No |
| 143 | IDH1 |  |  | 6741 | 0.576 | 0.3082 | No |
| 144 | DUT |  |  | 6839 | 0.539 | 0.3019 | No |
| 145 | TFB1M |  |  | 6848 | 0.535 | 0.3023 | No |
| 146 | UTY |  |  | 7034 | 0.475 | 0.2891 | No |
| 147 | APOC1 |  |  | 7046 | 0.471 | 0.2892 | No |
| 148 | MATN2 |  |  | 7154 | 0.435 | 0.2818 | No |
| 149 | RBBP4 |  |  | 7160 | 0.432 | 0.2823 | No |
| 150 | CREB1 |  |  | 7221 | 0.412 | 0.2785 | No |
| 151 | HIST1H3F |  |  | 7269 | 0.394 | 0.2757 | No |
| 152 | TSPAN8 |  |  | 7315 | 0.378 | 0.2730 | No |
| 153 | PRKG2 |  |  | 7360 | 0.364 | 0.2703 | No |
| 154 | ELAVL3 |  |  | 7371 | 0.358 | 0.2702 | No |
| 155 | MMP13 |  |  | 7376 | 0.355 | 0.2706 | No |
| 156 | IL1R1 |  |  | 7459 | 0.325 | 0.2650 | No |
| 157 | CYP51A1 |  |  | 7468 | 0.322 | 0.2650 | No |
| 158 | DIAPH3 |  |  | 7628 | 0.259 | 0.2533 | No |
| 159 | TERF1 |  |  | 7762 | 0.207 | 0.2436 | No |
| 160 | SLC4A4 |  |  | 7779 | 0.198 | 0.2428 | No |
| 161 | CLCN1 |  |  | 8024 | 0.096 | 0.2243 | No |
| 162 | TUBB2A |  |  | 8294 | 0.002 | 0.2038 | No |
| 163 | LEP |  |  | 8310 | -0.001 | 0.2026 | No |
| 164 | MXD3 |  |  | 8312 | -0.002 | 0.2026 | No |
| 165 | FUS |  |  | 8367 | -0.024 | 0.1985 | No |
| 166 | REST |  |  | 8468 | -0.064 | 0.1910 | No |
| 167 | HOXD3 |  |  | 8563 | -0.107 | 0.1840 | No |
| 168 | UCP3 |  |  | 8593 | -0.126 | 0.1820 | No |
| 169 | FDFT1 |  |  | 8694 | -0.166 | 0.1747 | No |
| 170 | KIF22 |  |  | 8725 | -0.180 | 0.1728 | No |
| 171 | MRPL52 |  |  | 8770 | -0.196 | 0.1698 | No |
| 172 | COL15A1 |  |  | 8838 | -0.226 | 0.1651 | No |
| 173 | SCD |  |  | 8910 | -0.266 | 0.1602 | No |
| 174 | STEAP4 |  |  | 8922 | -0.272 | 0.1599 | No |
| 175 | NIPSNAP1 |  |  | 8930 | -0.279 | 0.1599 | No |
| 176 | POF1B |  |  | 9083 | -0.352 | 0.1490 | No |
| 177 | CHRDL1 |  |  | 9142 | -0.379 | 0.1453 | No |
| 178 | OTC |  |  | 9267 | -0.440 | 0.1367 | No |
| 179 | THY1 |  |  | 9288 | -0.451 | 0.1360 | No |
| 180 | LIG3 |  |  | 9367 | -0.492 | 0.1310 | No |
| 181 | GREM1 |  |  | 9466 | -0.546 | 0.1246 | No |
| 182 | EDG2 |  |  | 9472 | -0.549 | 0.1253 | No |
| 183 | FN3K |  |  | 9509 | -0.565 | 0.1236 | No |
| 184 | B3GAT3 |  |  | 9537 | -0.581 | 0.1227 | No |
| 185 | MTNR1A |  |  | 9719 | -0.679 | 0.1102 | No |
| 186 | NEURL |  |  | 9749 | -0.694 | 0.1093 | No |
| 187 | SC4MOL |  |  | 9882 | -0.772 | 0.1007 | No |
| 188 | ABHD3 |  |  | 9915 | -0.793 | 0.0998 | No |
| 189 | LIN7B |  |  | 9964 | -0.822 | 0.0977 | No |
| 190 | SPA17 |  |  | 9994 | -0.838 | 0.0971 | No |
| 191 | PTPRO |  |  | 10017 | -0.857 | 0.0971 | No |
| 192 | SNX5 |  |  | 10150 | -0.930 | 0.0888 | No |
| 193 | SPTBN4 |  |  | 10586 | -1.232 | 0.0580 | No |
| 194 | PER3 |  |  | 10646 | -1.279 | 0.0559 | No |
| 195 | SLC29A1 |  |  | 10768 | -1.365 | 0.0493 | No |
| 196 | MSH3 |  |  | 11027 | -1.592 | 0.0327 | No |
| 197 | TNNI3 |  |  | 11038 | -1.605 | 0.0351 | No |
| 198 | SFXN1 |  |  | 11053 | -1.618 | 0.0371 | No |
| 199 | PET112L |  |  | 11186 | -1.734 | 0.0304 | No |
| 200 | DKK3 |  |  | 11306 | -1.828 | 0.0248 | No |
| 201 | SLC35C2 |  |  | 11388 | -1.919 | 0.0223 | No |
| 202 | WISP2 |  |  | 11475 | -2.007 | 0.0196 | No |
| 203 | IGFBP5 |  |  | 11829 | -2.352 | -0.0028 | No |
| 204 | ATP6V1E1 |  |  | 11864 | -2.389 | -0.0007 | No |
| 205 | TRIB3 |  |  | 11900 | -2.438 | 0.0013 | No |
| 206 | THBS2 |  |  | 11978 | -2.536 | 0.0003 | No |
| 207 | EDNRA |  |  | 12239 | -2.899 | -0.0140 | No |
| 208 | SLC22A18 |  |  | 12251 | -2.912 | -0.0092 | No |
| 209 | LRIG1 |  |  | 12253 | -2.914 | -0.0036 | No |
| 210 | LUM |  |  | 12475 | -3.221 | -0.0143 | No |
| 211 | DKK2 |  |  | 12563 | -3.362 | -0.0144 | No |
| 212 | TAC1 |  |  | 12571 | -3.372 | -0.0085 | No |
| 213 | CRIP1 |  |  | 12584 | -3.406 | -0.0028 | No |
| 214 | PFDN5 |  |  | 12640 | -3.509 | -0.0002 | No |
| 215 | COL6A3 |  |  | 12657 | -3.535 | 0.0054 | No |
| 216 | CLEC3B |  |  | 12963 | -4.370 | -0.0095 | No |
| 217 | FMOD |  |  | 13009 | -4.484 | -0.0043 | No |
| 218 | GFRA1 |  |  | 13018 | -4.523 | 0.0038 | No |
| 219 | DHCR24 |  |  | 13061 | -4.682 | 0.0097 | No |
| 220 | THBS4 |  |  | 13174 | -5.197 | 0.0111 | No |
Table: GSEA details [plain text format]

  

Fig 2: GAY\_YY1\_DN: Random ES distribution      
 Gene set null distribution of ES for **GAY\_YY1\_DN**
